# An International Summit in Human Genetics and Genomics: Empowering clinical practice and research in developing countries

**DOI:** 10.1002/mgg3.599

**Published:** 2019-02-20

**Authors:** Manjit Kaur, Donald W. Hadley, Maximilian Muenke, P. Suzanne Hart

**Affiliations:** ^1^ National Human Genome Research Institute National Institutes of Health Bethesda Maryland

## Abstract

To help fill the knowledge gap in human genetics and genomics, an International Summit (IS) in Human Genetics and Genomics was conceived and organized by the National Human Genome Research Institute (NHGRI) of the National Institutes of Health (NIH) as a 5‐year initiative, from 2016 to 2020. In its first 3 years, 71 professionals from 34 countries received training.

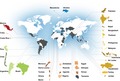

As mortalities from more common diseases and disorders decline, genetic disorders and congenital birth defects consume a disproportionate amount of resources for health and medical care, especially in low‐middle–income countries (LMICs) where >90% of the ~7 million babies born/year with a genetic disorder live (Verma & Puri, 2015). The birth prevalence of birth defects in LMICs is 20% higher than in high‐income countries, due to higher prevalence of specific conditions like hemoglobin disorders, G6PD deficiency, oculocutaneous albinism, Down syndrome, and neural tube defects. Seventy percent of these birth defects can be prevented, treated, or managed. However, many LMICs lack resources and expertise in genetics and genomics which impacts the provision of care and limits the training in this field. The double burden of disease and poverty takes a toll on the welfare of these nations. To help fill the knowledge gap in human genetics and genomics, an International Summit (IS) in Human Genetics and Genomics was conceived and organized by the National Human Genome Research Institute (NHGRI) of the National Institutes of Health (NIH) as a 5‐year initiative, from 2016 to 2020 ( https://www.InternationalSummit).

The goals of the IS are (a) to communicate advances in genomic science, (b) to identify and fill the knowledge gap in genetics and genomics and their related technologies, and (c) to promote genomic research and medicine, through international cooperation and collaboration. Here, we inform on the funding of the IS, its format, challenges identified by participants, and its early successes.

## FUNDING

Being a trans‐NIH initiative, funding was made available by multiple Institutes and Centers (ICs) at NIH (Table 1) and by contributions made to the Foundation for the NIH. Each sponsored participant received travel costs, lodging (double occupancy), and a daily stipend for meals and incidentals. Financial support was crucial for their attendance at the IS.

**Table 1:** NIH Institutes and Outside Organizations Providing Funding for the International Summit

National Institutes of Health (NIH) Institutes and Centers	Outside Organizations
Fogarty International Center (FIC)	American Dental Association Foundation
National Center for Advancing Translational Sciences (NCATS)	American Society of Human Genetics and Genomics
National Cancer Institute (NCI)	Bill & Melinda Gates Foundation
National Eye Institute (NEI)	Mayo Clinic
National Human Genome Research Institute (NHGRI)	March of Dimes Foundation
National Heart, Lung, and Blood Institute (NHLBI)	Medtronic
National Institute of Allergy and Infectious Diseases (NIAID)	NIH Foundation
*Eunice Kennedy Shriver* National Institute of Child Health and Human Development (NICHD)	Sequenom
National Institute on Drug Abuse (NIDA)	
National Institute on Deafness and Other Communication Disorders (NIDCD)	
National Institute of Dental and Craniofacial Research (NIDCR)	
National Institute on Minority Health and Health Disparities (NIMHD)	
National Institute of Neurological Disorders and Stroke (NINDS)	
National Institute of Nursing Research (NINR)	

*Note*.

NEI: National Eye Institute; NHGRI: National Human Genome Research Institute; NIDCR: National Institute of Dental and Craniofacial Research; NIH: National Institutes of Health; NINR: National Institute of Nursing Research.

## ELIGIBILITY AND APPLICATION REQUIREMENTS FOR PARTICIPANTS

Healthcare professionals across the health continuum from LMICs applied with a resume/curriculum vitae and a cover letter indicating: WHY they wanted to attend the Summit, WHAT impact the Summit would have on their careers, HOW the knowledge gained would be used to address the public health challenges specific to their nation, and a commitment for continued evaluation over 5 years. Three letters of recommendation were required indicating their professional abilities, enthusiasm and verification of employment and/or training status.

Applications were reviewed by a selection committee comprised of at least four individuals. Additional reviews were done by the sponsoring Institutes. Both a primary and a secondary list of candidates was prepared, ensuring a balance of scientific/health/clinical disciplines, countries, gender, preferred advanced training etc.

**Figure 1:**
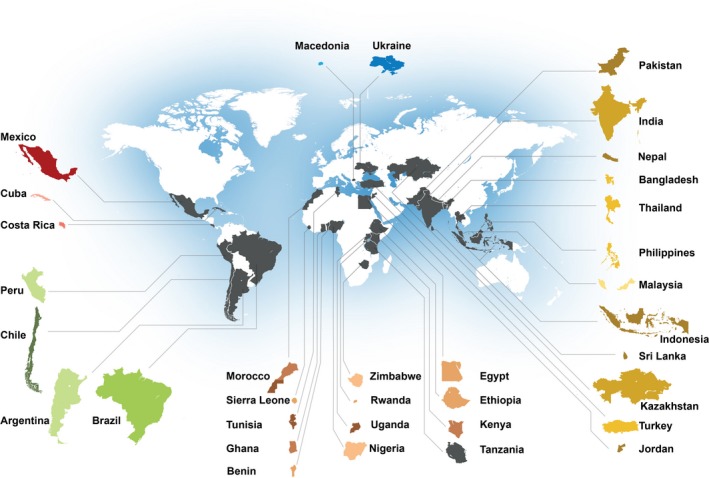
International summit countries

**Figure 2:**
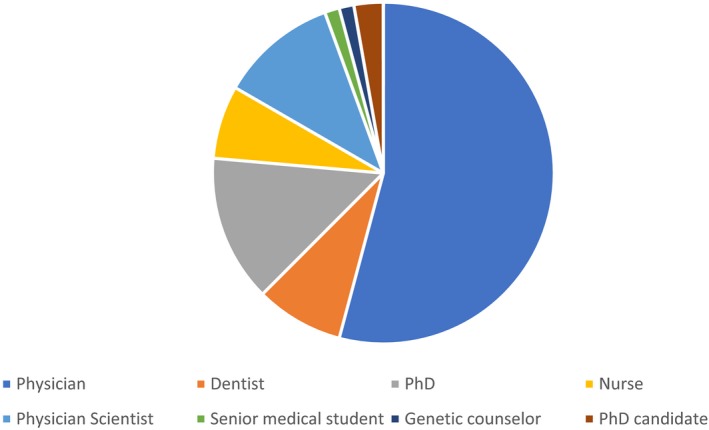
Background of participants

Between 2016 and 2018, 71 professionals from 34 countries received training (Figure 1). The background of these individuals is shown in Figure 2. While the majority (70%) of participants were clinicians, over 30% of the participants had a strong research focus.

## CURRICULUM

In 2018, the curriculum included 65 speakers that presented 70 didactic lectures across a broad spectrum of genetics and genomics, a hands‐on bioinformatics workshop, grant writing and reviewing session, a patient panel, and fieldtrips to various organizations (Children's National Medical Center, GeneDx, Johns Hopkins University, Kennedy Krieger Institute, NIH Intramural Sequencing Center, State of Maryland Newborn Screening Laboratory, and Washington Hospital Center). Visits to these facilities helped participants understand the spectrum of experts, infrastructure, and systems involved in the provision of genetic services and testing. Each year the patient panel has been a favorite among the participants. The grant writing session and visit to the State of Maryland Newborn Screening Laboratory were also favorites of the 2018 IS.

In addition to the above curriculum, there were 4.5 days dedicated to advanced training, tailored to the expressed needs and interests of the participants. For example, ophthalmologists were hosted by the National Eye Institute, dentists were hosted by the National Institute of Dental and Craniofacial Research and nurses were hosted by the National Institute of Nursing Research etc. Interested participants were provided with advanced training in clinical genomics and supercomputing utilization for whole‐genome analysis and interpretation. During this 1.5‐day next‐generation sequencing (NGS) training, participants learned to structure and execute common tasks needed for the interpretation of clinical whole genome and exome data. This included visual inspection of BAM files for variant confirmation, human phenotype ontology‐driven, whole‐genome analysis and Linux supercomputer programming.

Pre‐ and postsurveys were conducted each day to gauge knowledge, interest, and learning. The surveys were based on a 4‐point scale: 1 = strongly disagree, 2 = disagree, 3 = agree, and 4 = strongly agree. In 2016–2018, all components were rated as being informative and important for continued inclusion in subsequent IS. The IS curriculum is calibrated annually based on the cumulative feedback of past participants and the interests of the in‐coming participants (e.g., the addition of grant writing/reviewing in 2017 and of the Newborn Screening fieldtrip and NGS workshop in 2018).

Another highlight cited by many participants was the opportunity to network with investigators at NIH and surrounding institutions as well as among themselves. As noted under Early Successes, this has led to clinical and research collaborations, publications, shared resources, and the expansion of genetics/genomics in their countries.

## BARRIERS IDENTIFIED BY PARTICIPANTS

Participants were asked to identify barriers in their countries regarding the incorporation of genetics and genomics into clinical practice and research. The top three barriers identified were a lack of resources, both financial and human, a lack of appreciation of genetics in contributing to diseases in their countries and a lack of educational opportunities in genetics and genomics.

The IS empowered these participants to lower these barriers by the use of (a) strategies delivered by Dr. Carmen Padilla from the Philippines and a few others that included scientific, public and political awareness, and resource improvisation tactics. This provided participants with a potential roadmap; (b) data collection, documentation, and establishment of registries, which would demonstrate the impact of genetic disorders in their countries for engagement of health ministries; and (c) dissemination of knowledge delivered by experts in the field and by articulation and distribution of electronic copies of the lectures that can be delivered to different audiences. This will help fill the knowledge gap and improve the education and curriculum in genetics/genomics.

## EARLY SUCCESSES

Participants described the Summit as being “invaluable,” “magnificent,” “[making] an impact on my life and career” and “an opportunity of a lifetime.” All expressed gratitude for the opportunity to attend.

The early success and impact of the IS can be gauged through its two annual reports published in 2017 and 2018 (2016 outcomes, 2017 outcomes). Accomplishments include the establishment of birth defect registries in Indonesia, Nigeria, and Rwanda; the establishment or expansion of newborn screening in India, Indonesia, and Malaysia; expansion of genetic testing in Egypt, India, Indonesia, Nigeria, and Turkey; genetic counseling instituted or improved upon in Nigeria, Tanzania, and Malaysia; advocacy efforts to increase awareness are underway in Indonesia, Nigeria, and Tanzania where Health Ministries have also been engaged in discussion.

Several grants, research proposals, and articles were submitted or published by IS participants. Almost 200 articles (51—2016 outcomes; 147—2017 outcomes) have been published in their field of expertise. Many received or submitted grants to NIH or other funding institutions (24—2016 outcomes, 49—2017 outcomes).

Many collaborations (27—2016 outcomes; 51—2017 outcomes) were established with investigators at NIH or with other US‐based academic institutions and among themselves. New research and/or clinical projects (*n* = 43) were initiated and the course of ongoing research projects (*n* = 35) was influenced.

Several participants lectured as part of their curriculum on genetics and genomics at their institutions and attended and/or presented at professional/scientific conferences/meetings in their home countries or abroad. This is an important outcome as dissemination of knowledge was a specified goal of the IS.

In addition, several “Genetics and Genomic Medicine Around the World” articles were published by IS participants, including by Ariani, Soeharso, & Sjarif, 2017 (Indonesia), Balbuena & Teruel, 2017 (Cuba), Belhassan, Ouldim, & Sefiani, 2016 (Morocco), Guio et al., 2018 (Peru), Sukarova‐Angelovska & Petlichkovski, 2018 (Macedonia), and Vishnopolska et al., 2018 (Argentina).

The comprehensive list of accomplishments and information on the IS can be found on the IS website (https://www.genome.gov/27563951/an-international-summit-in-human-genetics-and-genomics/)

## CONCLUSION AND FUTURE

From its early success, it is evident that the IS is helping build capacity in research and medicine by filling the knowledge gap in genetics and genomics in LMICs, through international cooperation and collaborations. But, it is clear that this is not enough, as the cohorts are small (max *n* = 30) due to funding constraints. Attendance is also limited due to visa‐related and/or employment issues. This restricts the number of talented deserving healthcare professionals that can attend the IS and delays the integration and advancement of genetics and genomics in vulnerable populations, where it is needed the most. The IS has offered several avenues for further advancement and many participants have expressed interest in pursuing more formal genetics training in the United States.

The IS has gained momentum and in 2019 the IS will be held from August 28 to September 28. Over 200 professionals have applied for 30 positions.

Given our 3 years of experience with the IS, NHGRI is committed to developing a genetics/genomics certificate program for individuals from LMICs that would mirror training for our clinical and Ph.D. fellows. Individuals in the certificate program would take NHGRI graduate classes remotely. The program would also include development of a research proposal involving genetics and/or genomics to further demonstrate proficiency in the area. More details will be released soon, along with the application procedure and specific format/curriculum.

## CONFLICT OF INTEREST

None declared.
